# Evaluation of a bone morphogenetic protein 6 variant as a cause of iron loading

**DOI:** 10.1186/s40246-018-0155-5

**Published:** 2018-04-25

**Authors:** Cameron J. McDonald, Gautam Rishi, Eriza S. Secondes, Lesa Ostini, Daniel F. Wallace, Darrell H. G. Crawford, Hanlon Sia, Paul Clark, V. Nathan Subramaniam

**Affiliations:** 1 QIMR Berghofer Medical Research Institute, Brisbane, Australia; 2 Institute of Health and Biomedical Innovation and School of Biomedical Sciences, Queensland University of Technology (QUT), 60 Musk Avenue, Kelvin Grove, Brisbane, Queensland 4059 Australia; 3 Faculty of Medicine and Biomedical Sciences, The University of Queensland, Brisbane, Australia; 4 ICON Cancer Care, Southport, Australia; 5 Princess Alexandra and Mater Hospitals, Brisbane, Australia

**Keywords:** Next-generation sequencing, Hemochromatosis, Causal variant, Iron overload, BMP6

## Abstract

**Background:**

Atypical iron overload without variation in the five clinically associated hereditary hemochromatosis genes is now recognized; however, their etiology remains unknown. Since the identification of iron overload in the bone morphogenetic protein 6 (*Bmp6*) knockout mouse, the search has been on for clinically pathogenic variants in the *BMP6* gene. A recent report proposes that variants in the pro-peptide region of *BMP6* are the underlying cause of several cases of iron overload. We performed targeted next-generation sequencing on three cases of atypical iron overload with Asian ethnicity and identified a p.Q118dup (aka p.E112indelEQ, p.Q115dup, p.Q118_L119insQ) variant in *BMP6*. The purpose of this study was to characterize the molecular function of the identified BMP6 variant. Molecular characterization by immunofluorescence microscopy and Western blotting of transfected cells, bioinformatics, and population analyses was performed.

**Results:**

In contrast to reports for other BMP6 pro-peptide variants in this region, our data indicates that this variant does not affect the function of the mature BMP6 protein.

**Conclusions:**

Our data suggest that assignment of disease causation in clinical cases of iron overload to pro-peptide variants in BMP6 should thus be treated with caution and requires biological characterization.

## Background

Hereditary hemochromatosis (HH) is relatively common in individuals of northern European descent, with the majority of cases caused by mutations in the *HFE* (hemochromatosis) gene [1, 2]. Conversely, mutations in *HFE* are rare in non-European populations, and mutations in other genes involved in the regulation of iron homeostasis are more common, such as hemojuvelin (*HFE2)*, hepcidin (*HAMP)*, transferrin receptor 2 (*TFR2*), and ferroportin (*SLC40A1*), collectively termed non-*HFE* HH [3]. Potentially, all regulators of iron homeostasis are functional gene candidates for non-*HFE* HH [4]. BMP6 (bone morphogenetic protein 6) is a key regulator of iron homeostasis [5]. *Bmp6* null mice have significant iron overload due to deficiency of the iron-regulatory hormone hepcidin [6, 7].

Under normal conditions, *Bmp6* expression is upregulated by increased iron and increases the expression of hepcidin which in turn limits iron absorption and recycling [8]. A recent report associated heterozygosity for three variants p.P95S, p.L96P, and p.Q113E in the BMP6 pro-peptide region with nine clinical cases of unexplained atypical iron overload [9]. The functional link between BMP6 and iron overload remains to be explained, however, and further validation is necessary to better assess causality of these variants [10]. In addition, p.E112Q and p.R257H have also been associated with more severe iron overload in patients with *HFE* mutations [11]. Here, we report on three clinical cases of unexplained atypical iron overload carrying an amino acid insertion within the same region of the BMP6 pro-peptide.

## Results

**Fig. 1:**
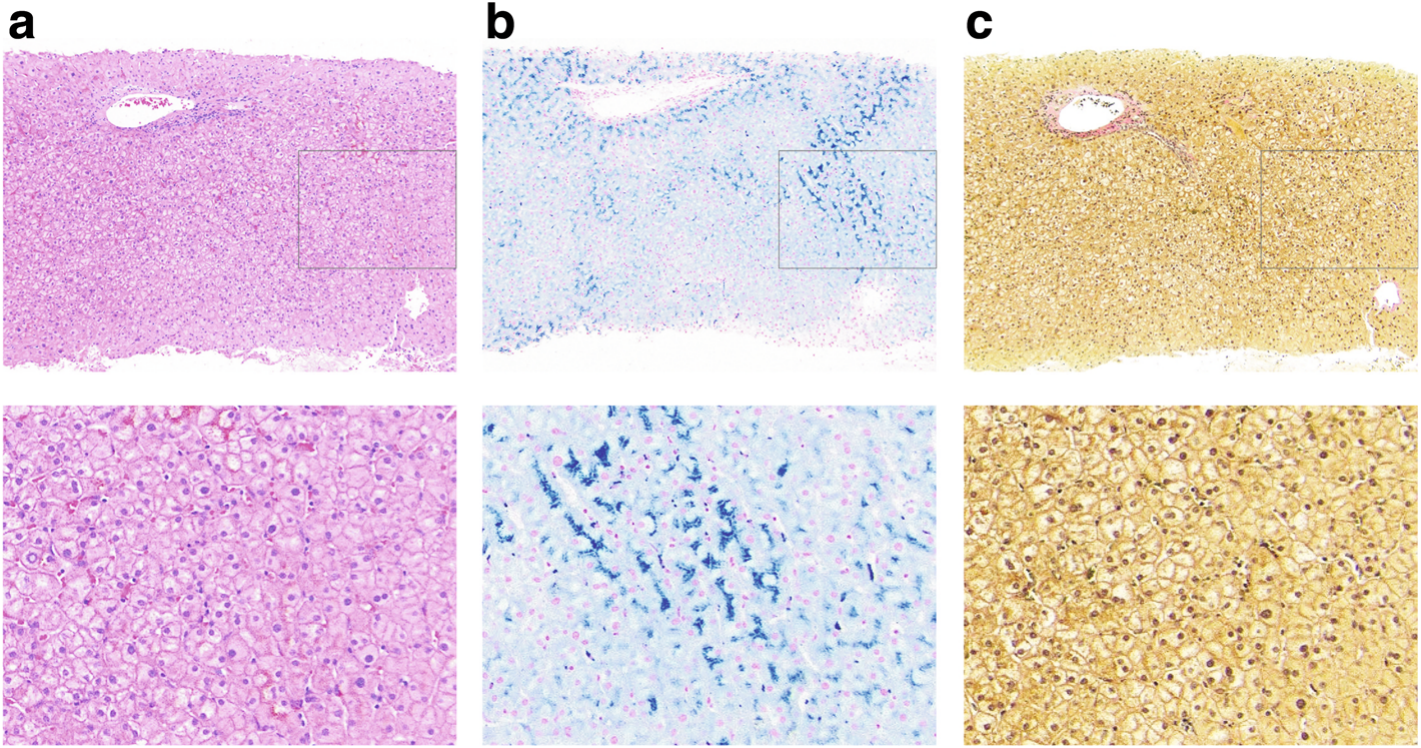
Liver biopsy histology of case 1. Histological sections stained with **a** H&E, **b** Perls, and **c** Van Gieson’s stain showing significant stainable iron, without pathology

Three cases are reported in this study; in each case, *HFE* genotyping was negative, and liver specialist assessment was undertaken. Case 1 was a 62-year-old male who over the past 7 years had raised serum ferritin (SF) ranging between 1261 and 1675 μg/L (normal range 30–300 μg/L), with transferrin saturation (TS) 40 to 53% (normal range 20–55%). His alanine aminotransferase (ALT), aspartate aminotransferase (AST), and gamma-glutamyl transferase (GGT) levels all remained normal during this time. Liver biopsy revealed grade 4 stainable iron in hepatocytes, with accentuation of iron in the pericanalicular region (Fig. 1). No other pathology was noted. In case 2, a male aged 51 who did not consume any alcohol, presented with a SF of 1714 μg/L and TS of 34%. ALT, AST, and GGT were all normal although ultrasound revealed a diffusely echogenic liver suggesting fatty liver. Ferriscan identified a hepatic iron concentration of 44 μmol/g (normal range 3–33 μmol/g). In case 3, also male aged 62 presented with a SF of 1133 μg/L and TS of 90%. His ALT, AST, and GGT levels were mildly elevated. Liver biopsy demonstrated grade 1 iron with a hepatic iron content of 39 μmol/g and F1 fibrosis. Case 3’s pathophysiology is complex with positivity for α-thalassemia trait and hepatitis C virus (genotype 2).

Targeted next-generation sequencing revealed that all three patients were negative for mutations in the five currently clinically associated HH genes. Further analysis identified a tri-nucleotide duplication in *BMP6*, c.353-355dupAGC (NM_001718.4), resulting in the in-frame duplication of glutamine p.Q118dup (NP_001709.1), which was confirmed by Sanger sequencing. The nomenclature for this variant is noted in three different forms across various datasets: dbSNP rs771616962 refers to this variant as p.Q118_L119insQ, wANNOVAR, and the NHLBI Exome Sequencing Project (http://evs.gs.washington.edu/EVS/) (ESP) dataset as p.E112delinsEQ and Exome Aggregation Consortium database (ExAC) [12] as p.Gln115dup. While all of these references result in the same outcome, extension of the poly-Q stretch, the various designations may cause underestimation of its population frequency. This variant, or its pseudonyms, is not present in the 1000 Genomes database; however, it is present in the ESP (MAF = 0.016) and ExAC (MAF = 0.041).

**Fig. 2:**
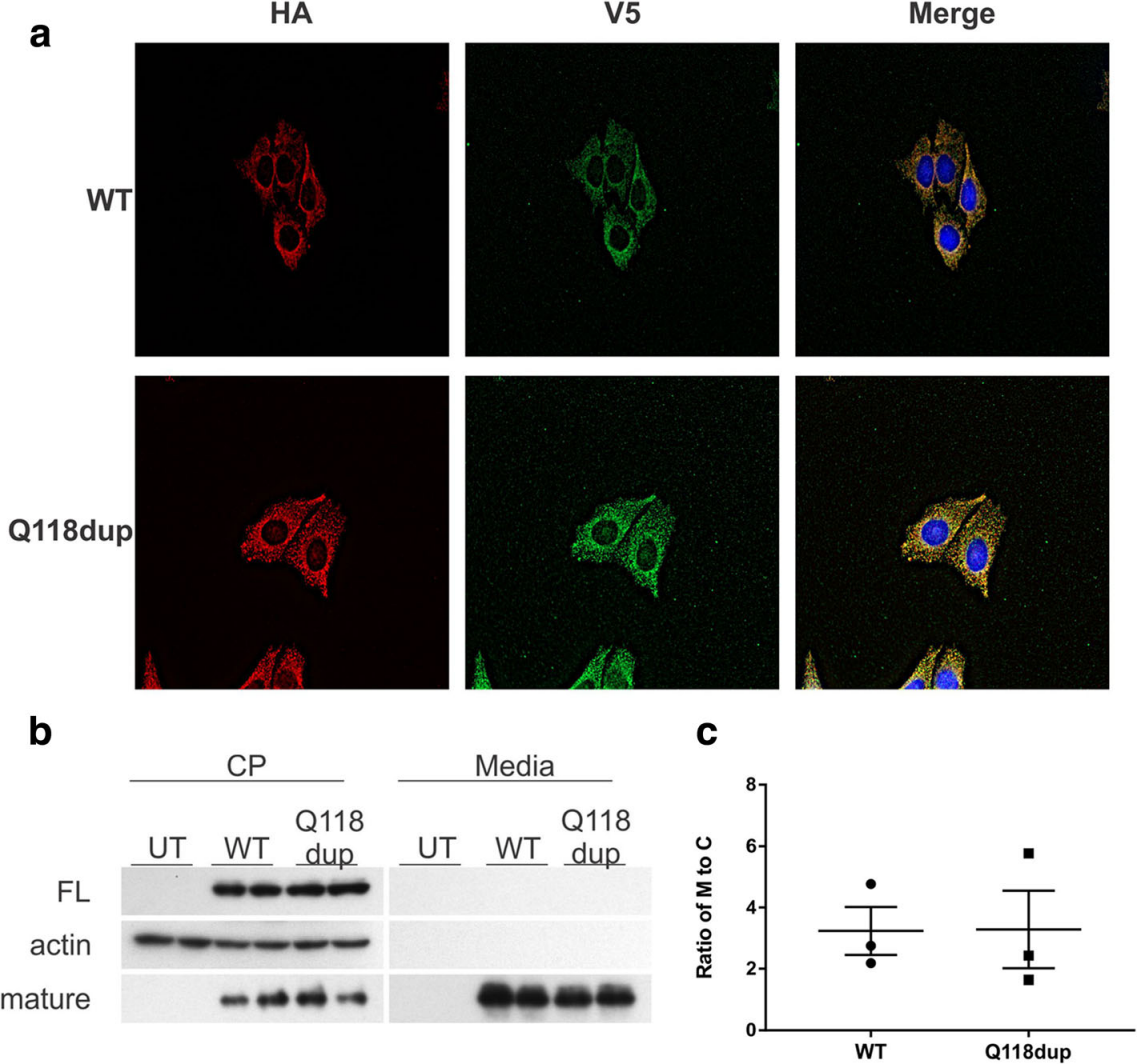
Cellular characterization of p.Q118dup BMP6 protein in CHO cells. **a** Representative immunofluorescence image of stably transfected CHO cells. The HA tag on the N-terminus of the pro-peptide, and the V5 tag on the C-terminus of the mature protein co-localize, and no difference in localization of the wildtype (WT) and p.Q118dup variant of BMP6 was observed. **b** Western blot identifying the full-length (FL) and mature BMP6 proteins in cell lysate (CP) and conditioned media (M) from untransfected (UT), wildtype (WT), and p.Q118dup (Q118dup) BMP6 transfected CHO cells. **c** Quantification of Western blot shown in **b** (*n* = 3). The ratio of mature proteins in conditioned media (M) to cell lysate (CP) shows no difference in protein secretion between WT and p.Q118dup BMP6 transfected cells

**Fig. 3:**
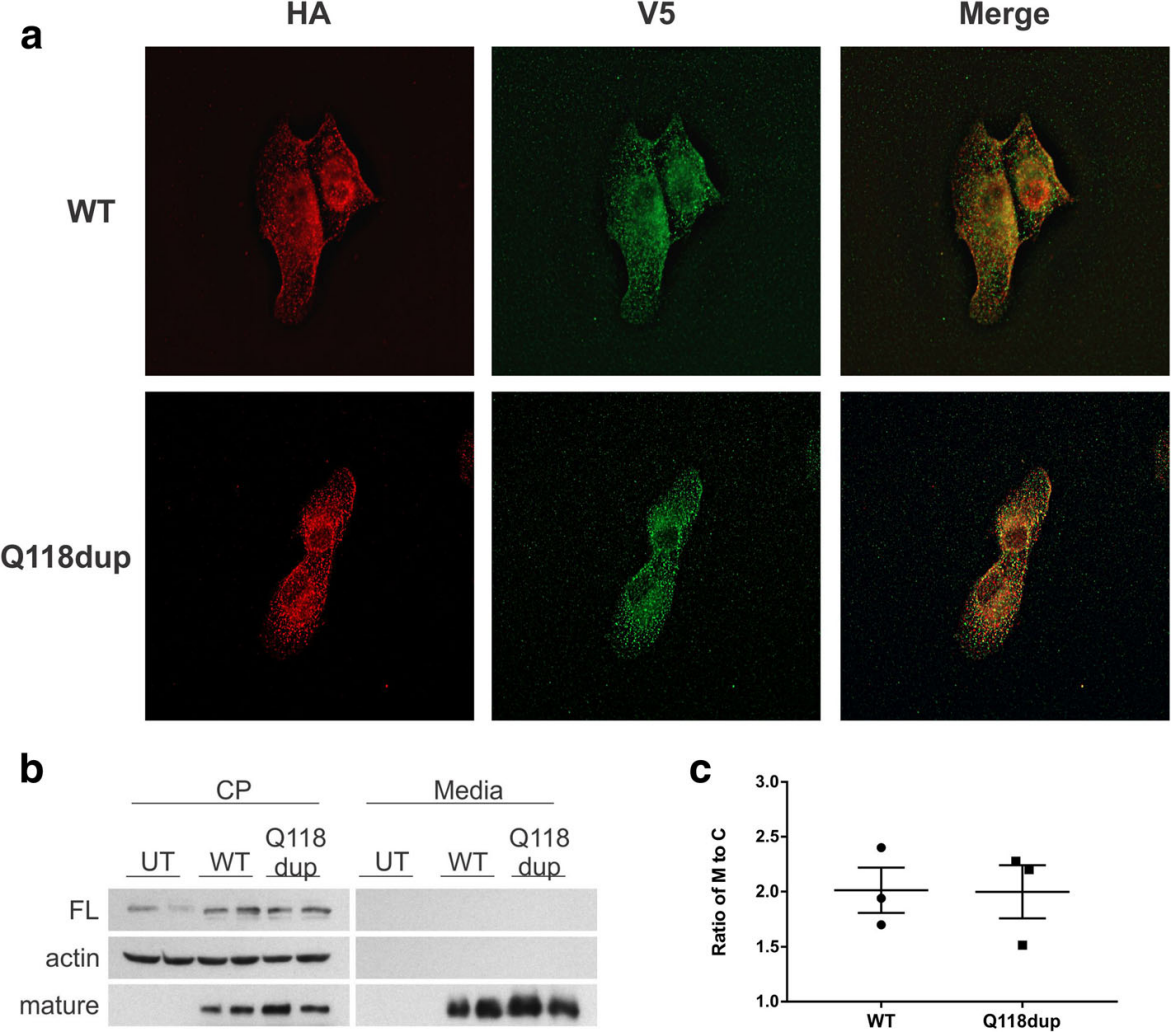
Cellular characterization of p.Q118dup BMP6 protein in a hepatic endothelial cell model, SK-HEP-1. **a** Immunofluorescence of stably transfected SK-HEP-1 cells. No difference in localization of the wildtype and p.Q118dup variant of BMP6 was observed. HA tag, N-terminus of pro-peptide; V5 tag, C-terminus of mature protein. **b** Western blot identifying the full-length (FL) and mature BMP6 proteins in cell lysate (CP) and conditioned media from untransfected (UT), wildtype (WT), and p.Q118dup BMP6 transfected SK-HEP-1 cells. **c** Quantification of Western blot shown in **b** (*n* = 3). The ratio of mature proteins in conditioned media to cell lysate shows no difference in protein secretion between WT and p.Q118dup BMP6 transfected cells

To investigate possible functional effects of the p.Q118dup variant on BMP6, immunofluorescence microscopy studies were conducted using BMP6 wildtype and p.Q118dup proteins with a pro-peptide N-terminus HA tag and a mature protein C-terminus V5 tag. We used both CHO cells and liver endothelial (SK-HEP-1) cells as a model for examining the molecular and functional effects of the p.Q118dup variant. CHO cells (Fig. 2a) and SK-HEP-1 cells (Fig. 3a) stably expressing these constructs showed some peri-nuclear Golgi localization of p.Q118dup pro-peptide; however, this was very mild and not consistent within all cells. No difference in localization was observed for the mature V5-tagged protein in either the CHO or endothelial cell models.

## Discussion

BMP6 is an important signaling molecule required for iron homeostasis [6, 8]. In response to increase in body iron levels *BMP6* levels increase, this results in an increase in BMP-SMAD signaling. The increased BMP_SMAD signaling then leads to an increase in hepcidin production. Recently, Daher and colleagues reported on three BMP6 pro-domain variants associated with iron overload and attributed the cause to reduced export of BMP6 [9]. We compared levels of the full-length and mature wildtype and mutant BMP6 protein in cell lysate and conditioned media (Fig. 2b, c). This revealed no difference in the capacity of cells to export p.Q118dup BMP6 protein compared to wildtype. Recent studies have suggested that the endothelial cells may be responsible for BMP6 production in response to iron [13]. Using an endothelial cell model, we show that there is no difference in the expression of the mature BMP6 protein for either the wildtype or p.Q118dup variant of BMP6 (Fig. 3b).

As population frequency can provide insight into the likelihood of a variant being benign, we investigated the frequency of p.Q118dup in the ExAC dataset (http://exac.broadinstitute.org/) which contains exome sequences from 60,706 unrelated individuals, and carrier rates were then calculated using the Hardy-Weinberg equation. This analysis reveals that in East Asians, p.Q118dup has a carrier rate of 1 in 3 (MAF = 0.191), with other ethnicities also showing lower though still significant carrier rates, confirming the experimental result that this variant is unlikely to affect gene function. Finally, we examined the classification of the p.Q118dup variant according to the “standards and guidelines for the interpretation of sequence variants” as set out by the American College of Medical Genetics and Genomics [14]. Based on the above data plus bioinformatic functional impact prediction using several tools available through ANNOVAR, it confirmed a categorization of the BMP6 pro-peptide p.Q118dup variant as benign.

## Conclusions

The data from this study suggest no functional relationship between the p.Q118dup BMP6 pro-peptide variant and iron overload. Recognition of this variant as non-deleterious and not causative is notably important for this specific variant, as cursory literature and database analysis may easily lead to the alternate and incorrect assumption that this variant is likely causative of the disease.

Firstly, the alternative nomenclature of p.E112indelEQ (which while technically incorrect is the annotation returned by several common sequence alignment programs) places this variant at the same location as the p.Q113E variant recently reported by Daher et al. [9] to be deleterious and the cause of atypical iron overload in two members of a single family. Secondly, the absence of p.Q118dup from the two most commonly known variant data sets, dbSNP and 1000 Genomes, erroneously suggests that this variant is novel and thus is not carried in the healthy population. Finally, using CHO and hepatocyte endothelial cell models, we showed no difference in the localization or secretion between the wildtype and p.Q118dup BMP6 variant.

These data demonstrated no relationship between *BMP6* variants and atypical iron overload and provided no functional evidence of causality for the identified variant. Furthermore, the analysis raises uncertainty about the population prevalence of *BMP6* variants and their potential role in iron overload conditions. This has important clinical implications, for monitoring and treatment, and should lead to pause before suggesting genetic and family counseling based on *BMP6* variation. Atypical, non-HFE-related iron overload remains an important question; however, despite prior literature and the appeal of its underlying biological plausibility, the genetic causes of some forms of HH appear elusive. Further research will be required to uncover the potential genetic causes of these atypical iron overload disorders.

## Methods

### Patients and consent

This study was approved by and performed in accordance with the ethical standards of the QIMR Berghofer Human Research Ethics Committee and with the Helsinki Declaration of 1975, as revised in 1983. Informed and written consent was obtained for the studies described in this report.

Cases of clinically identified atypical iron overload of unknown cause were referred to our laboratory for sequencing by primary clinicians. Cases were required to demonstrate persistently (two or more occasions) abnormal iron indices (including significantly elevated serum ferritin > 1000 μg/L for males and > 700 for females) and to be negative for p.C282Y homozygosity. Referring clinicians were also requested to undertake a detailed iron metabolism-specific patient history for other possible attributing causes, such as high alcohol consumption. In total, 31 referred patients have been sequenced to date at our center [15].

### Next-generation sequencing

Sequencing of genomic DNA using an iron-associated gene panel, covering 39 genes and 11 promoters associated with iron overload or anemia, was performed as previously described [15]. Samples were sequenced to an average of 400-fold coverage using an Ion Torrent PGM, with post run QC/QA filtering performed using Torrent Suite (v3.6; Life Technologies). Sequences were mapped to the Human Genome (HG19) and variants identified using the Torrent Variant Caller (version 3.6.59049 with Germ Line - Low Stringency configuration). Identified variants were annotated using the wANNOVAR tool [16], and candidate mutations confirmed by Sanger sequencing.

### BMP6 expression constructs

Constructs containing double-tagged human *BMP6* coding sequence were synthesized and validated by BioMatik (Delaware, USA). Constructs were generated for both the wildtype and p.Q118dup sequence and contained a hemagglutinin (HA)—tag inserted between amino acids 23 and 24 (N-terminus of the pro-protein) and a V5-tag at the carboxyl-terminus of the mature protein.

### Transfections

Chinese hamster ovary (CHO) cells and the hepatic endothelial cell line, SK-HEP-1 [17] (HTB-52, ATCC, Manassas, Virginia), were transiently transfected with the *BMP6* expression constructs by reverse transfection using Viafect (Promega, Alexandria, NSW, Australia) according to the manufacturer’s instructions. Stably expressing cells were obtained by placing transfected cells under selection with puromycin at 10 μg/ml (CHO cells) or 1 μg/ml (SK-HEP-1 cell).

### Immunofluorescence

Transfected cells were plated on glass coverslips to a confluency of 50–70% for 24 h. Cells were washed twice in PBSCM (phosphate buffered saline with 1 mM CaCl_2_, 1 mM MgCl_2_) and then fixed (in methanol at − 20 °C for 5 min for CHO cells then at room temperature (RT) for 10 min and in 3% paraformaldehyde for SK-HEP Hep-1 cells which were then permeabilized using 0.1% saponin in PBSCM for 15 min). Primary antibodies were then applied: anti-HA at 1:10 (12CA5, tissue culture supernatant) and anti-V5 at 1:3000 (Merck-Millipore). After three PBSCM washes, cells were incubated in Alexa Fluor donkey secondary antibodies: anti-mouse 488/594 and anti-rabbit 594/488 (Invitrogen). Following three final PBSCM washes, coverslips were mounted in Prolong Gold anti-fade mounting media (Invitrogen) and imaged by microscopy at × 63 magnification using a Zeiss Axioscope NLO-780 or a Leica TCS SP5. Z stacks were imaged for each focal point, and the final images were processed using the in-built deconvolution algorithms in the Zen software. The immunofluorescence studies were performed at least three times for each cell line.

### Western blotting

Conditioned media from transfected cells were precipitated with 10% trichloroacetic acid overnight at 4 °C, and the precipitate was washed with cold acetone and resuspended in SDS sample buffer (SB) (50 mm Tris pH 6.8, 2% SDS, 10% glycerol, 0.1% bromophenol blue, 5% β-mercaptoethanol). Cell lysates were also resuspended in SDS SB, and all samples were loaded onto a 12% SDS-PAG and electrophoresed. Following transfer onto 0.2 μm nitrocellulose (Bio-Rad), blots were blocked for 3 h at RT in Western blocking buffer WBB (10% skim milk powder in 0.5% Tween-20 in TBS (TBST)). Primary anti-V5 (2.5 ng/ml) or anti-HA (1:100) diluted in WBB was applied overnight at 4 °C. Following three washes in TBST, blots were incubated with secondary anti-mouse horseradish peroxidase (1:10,000 in WBB) for 1 h at RT. Blots were washed extensively with TBST, Luminata Forte Western Chemiluminescent HRP Substrate (Merck-Millipore) was applied for 5 min, and blots were then exposed to film. Western blotting was repeated three times for each cell line and quantified. Band volume and density were quantitated using ImageJ software (National Institutes of Health, Maryland, USA).

## Abbreviations

ALT: Alanine aminotransferase
AST: Aspartate aminotransferase
BMP: Bone morphogenetic protein
ESP: NHLBI Exome Sequencing Project 6500 database
ExAC: Exome Aggregation Consortium database
GGT: Gamma-glutamyl transferase
SF: Serum ferritin
TS: Transferrin saturation
